# Genetic Polymorphism of rs13306146 Affects *α_2A_AR* Expression and Associated With Postpartum Depressive Symptoms in Chinese Women Who Received Cesarean Section

**DOI:** 10.3389/fgene.2021.675386

**Published:** 2021-07-07

**Authors:** Kai Ming Duan, Chao Fang, Si Qi Yang, Shu Ting Yang, Ji Dong Xiao, Huang Chang, Guo Xin Lin, Liang Bin Zhang, Ming Chao Peng, Zhao Qian Liu, Sai Ying Wang

**Affiliations:** ^1^Department of Anesthesiology, Third Xiangya Hospital of Central South University, Changsha, China; ^2^Hunan Cancer Hospital, The Affiliated Cancer Hospital of Xiangya School of Medicine, Central South University, Changsha, China; ^3^Department of Ultrasonography, Third Xiangya Hospital of Central South University, Changsha, China; ^4^Department of Clinical Pharmacology, Xiangya Hospital, Central South University, Changsha, China; ^5^Hunan Key Laboratory of Pharmacogenetics, Institute of Clinical Pharmacology, Central South University, Changsha, China

**Keywords:** α_2_-adrenoceptors, postpartum depression, single nucleotide polymorphism, miRNA, expression

## Abstract

Postpartum depressive symptom (PDS) is a common psychological and mental disorder after giving birth. Our previous studies showing the application of dexmedetomidine, an α_2_-AR agonist, can significantly improve maternal sleep, as well as relieve and reduce the incidence of PDS. This study investigated the association between α_2__*A*_AR gene polymorphisms and PDS. A total of 568 cesarean section patients were enrolled; the incidence of PDS is 18.13% (103 with PDS, 465 with non-PDS). The Edinburgh Postpartum Depression Scale score ≥10 was used to diagnose PDS at 42 days after delivery. The single-nucleotide polymorphisms of α_2A_R were sequenced by pyrosequencing. The effect of rs13306146 A > G polymorphism on α_2A_R transcription and the regulation of miR-646 on α_2A_R expression were assessed by dual luciferase reporter assays or gene transfection. Increased stress during pregnancy, poor relationship between mother-in-law and daughter-in-law, spousal relationship, domestic violence, antenatal depression, self-harm ideation, and stressful life events were all associated with increased PDS incidence (*p* < 0.05). The logistic regression analysis found that the α_2A_AR rs13306146 polymorphism was associated with PDS after adjusting confounding variables. The transcriptional function of the α_2A_AR rs13306146 A allele was decreased compared with the G allele, and the α_2A_AR expression level was correspondingly decreased (*p* < 0.05), as the strongest binding ability of miR-646 to the α_2A_AR rs13306146 AA genotype. The effect of α_2A_AR rs13306146 A > G polymorphism may change the binding ability of miR-646 at the 3′UTR of the α_2A_AR gene, affecting the expression of α_2A_AR. This study supports the involvement of the norepinephrine system in the pathogenesis of PDS. Genotypes of α_2A_AR may be novel and useful biomarkers for PDS.

## Introduction

Postpartum depressive symptoms (PDS) are common among women, with an incidence of between 10 and 20% (Gavin et al., 2005). PDS can have a serious and long-term negative effect on mothers, including on both marital and mother–infant relationships. Severe PDS can have devastating consequences, including maternal self-harm and suicide (Lazinski et al., 2008; Pinheiro et al., 2008). The pathogenesis of PDS is complex, as indicated by the wide array of PDS-related risk factors and the dynamic physiological changes occurring in women following birth. Recent data shows a wide complexity of central and systemic processes that may underpin mood dysregulation, which seem to interact with the dynamic physiology of parturition (Anderson and Maes, 2013, 2014; Duan et al., 2018).

Data accumulated over many decades indicates a role for the central noradrenergic nervous system in mood dysregulation, with preclinical data showing chronic stress to lead to an imbalance in the structure and function of the central adrenergic nervous system (Itoi and Sugimoto, 2010). Stress during pregnancy and the perinatal period is a major PDS risk factor, with raised levels of plasma norepinephrine (NE) evident in PDS, vs. controls (Xie et al., 2018). Doornbos et al. (2008) have proposed that postpartum women are more sensitive to stress, with the plasma NE metabolite, 4-hydroxy-3-methoxyphenyl-glycol (HMPG), increased in PDS, being an indicant of heightened arousal. The locus coeruleus is the main source of central NE, which significantly regulates stress *via* effects within the brain as well as *via* the regulation of the sympathetic nervous system (Bangasser et al., 2016; Gu et al., 2016). Stress-induced increases in locus coeruleus NE modulate function in diverse brain regions, including in the brain stem, hypothalamus, cerebellum, amygdala, and prefrontal cortex. Preclinical data clearly shows chronic stress-induced depression to involve alterations in locus coeruleus NE release (Kitayama et al., 2004; Seki et al., 2018), which in the lipopolysaccharide (LPS) preclinical model can be prevented by blocking α_1_ central noradrenergic receptors (Sekio and Seki, 2015). Such data indicates central noradrenergic neuronal activity to be an antidepressant treatment target, with antidepressants shown to regulate the activity of locus coeruleus NE neurons and NE levels in rats (Kawahara et al., 2007). α_2_-Adrenoceptors (AR) are important in the regulation of central NE neurons, being distributed in both presynaptic and postsynaptic membranes, with presynaptic α_2_-AR important to the modulation of central NE release (Millan et al., 2000). Stress inhibits central α_2_-AR function, with the intraventricular injection of the α_2_-AR agonist, clonidine, improving depression-like behavior in preclinical models (Weiss and Simson, 1988). The clinical relevance of such preclinical data is supported by our previous studies showing that the application of dexmedetomidine, an α_2_-AR agonist, during the perinatal period can significantly improve maternal sleep, as well as relieve, and reduce the incidence of, PDS (Yu et al., 2019).

The α_2A_Rs are divided into three main subtypes, namely, α_2A_AR, α_2B_AR, and α_2c_AR, with the presynaptic α_2A_AR being the main inhibitory adrenergic neuron autoreceptor (Trendelenburg et al., 1993; Bylund et al., 1994). Both preclinical and clinical studies indicate that α_2A_AR is intimately linked to depressive symptomatology. Depressed suicidal patients show significantly altered α_2A_AR levels in the forebrain, hippocampus, and hypothalamus (Meana et al., 1992). The density and affinity of platelet α_2A_AR are increased in depressed, which are reduced following treatment with antidepressant drugs or electrical shock (Smith et al., 1983). Butler and Leonard (1986) also reported an increased density of lymphocyte adrenergic receptors in PDS patients, which decreased following 6 weeks of antidepressant treatment. Such data show that alterations in systemic α_2A_AR levels and function may be evident in depression. The knockout of α_2A_AR in mice leads to a reduced motor coordination capacity as well as increased anxiety and depressive-like symptoms (Schramm et al., 2001; Lähdesmäki et al., 2002). Preclinical data indicate that antidepressant treatment efficacy is mediated by treatment-induced decreases in α_2A_AR, following chronic stress-induced elevations in the presynaptic α_2A_AR that arise from stress-mediated rise in central NE (Wang B. et al., 2017). Overall, such data would suggest that α_2A_AR is an important regulator of depressive responses to chronic stress, with relevance to PDS pathobiology. As well as stress and antidepressants, the central noradrenergic nervous system can also be regulated by genetic background (Zhang et al., 2014; Figueiredo et al., 2015). Previously, we showed that SNPs of the metabolic enzymes monoamine oxidase (MAO)-A and catechol-*O*-methyltransferase (COMT) which regulates NE concentration in the synaptic cleft of central adrenergic neurons are associated with PDS incidence (Ma J. et al., 2019). Doornbos et al. (2009) showed PDS to be related to MAO-A SNPs, with depression before and 6 weeks after delivery, which is significantly increased in women with dual low activity MAO-A and COMT alleles. A study found that the N251K variation of *α_2A_AR* has been linked to suicidal ideation in depressed patients (Sequeira et al., 2004), but the association between *α_2A_AR* polymorphisms and PDS is still unknown.

We consider the variations in central α_2_-AR level and function as an aspect of postpartum depressive symptomatology. The present study aimed to investigate the role of α_2A_AR polymorphisms in PDS, by looking at the selected SNPs of rs521674 and rs13306146 polymorphisms of α_2A_AR and their association with PDS.

## Materials and Methods

### Participants

This study enrolled 568 expectant parturients in Third Xiangya Hospital from June 2017 to December 2019. This project was approved by the ethics committee of the Third Xiangya Hospital of Central South University with a registration number of S155. All participants provided written informed consent before entering the study.

Inclusion criteria were as follows: American Society of Anesthesiologists (ASA) grade II; pregnant women aged at least 18 years, and with 28 weeks or greater gestation; voluntary participation agreement; and the ability to communicate with others. Exclusion criteria were as follows: the history of serious mental diseases or psychotropic drug utilization in the past month; complications arising from severe dysfunction of other important organs; and high surgical risk or life-threatening situation occurring during the operation (Pinsonneault et al., 2013; Wang S.-Y. et al., 2017).

### Collection of General Data, Clinical Data, and Samples of Parturient–Mothers

Basic personal data and clinically relevant data were acquired in detail, including age; artificial or natural pregnancy; birth experience; gestational age; whether singleton or not; severe, moderate, or mild stress during pregnancy (Chen et al., 1991; Zorumski et al., 2013); good, moderate, or poor marital relationship; the relationship between mother-in-law and daughter-in-law; domestic violence during pregnancy; education; monthly family income; antenatal depressive symptoms; self-harm ideation or not; and the history of stressful life events. Also, 5 ml of venous blood was taken from pregnant women, marked with the name and serial number in the anticoagulant vessels containing ethylenediaminetetraacetic acid (EDTA), then stored in the refrigerator at −20°C.

### Implementation of Maternal Surgery, Postpartum Follow-Up, and Determination of Outcome Indicators

All the pregnant women were admitted to the operating room after preoperative preparation. Monitoring included electrocardiography (ECG), noninvasive blood pressure, heart rate, and pulse oxygen saturation. Oxygen was administered by clear nasal catheter until delivery at a rate of 2 l/min. All the expectant women received combined spinal and epidural anesthesia. After the anesthesia effect met the operation conditions, the obstetrician performed the lower-segment cesarean section. The vital signs of the patients were closely observed during the operation. After fetal delivery, a decision was made as to whether to transfer the newborn to the neonatal intensive care unit, which was dependent upon the maternal condition and neonate features. After the operation, the parturients were returned to the ward.

The Chinese validated version of the Edinburgh Postpartum Depression Scale (EPDS) (Cox et al., 1987) was used to evaluate PDS on the first day before delivery and the 42nd day after delivery. An EPDS score ≥ 15 was used to denote prenatal depression, and an EPDS score ≥ 10 was used to diagnose PDS at 42 days after delivery (Lee et al., 1998; Falah-Hassani et al., 2017). The assessment of self-harm ideation utilized the last EPDS item when administered on the day before the operation, with parturients who gave a response of “yes, quite often” “sometimes” or “seldom” being categorized as “yes,” with only a response of “never” being classified as “no”(Ma J.-H. et al., 2019).

### Detection of SNP Locus of α_2_AR Gene in Parturient Women

(1) Acquisition of DNA specimens. All expectant women had a 5-ml sample of venous blood collected in a test tube containing EDTA. The procedure followed instructions of the DNA extraction kit, whereby after thawing, the DNA of the blood sample was extracted through the steps of cleavage, centrifugation, incubation, concussion, filtration, and dissolution. Following this procedure, the concentration and purity of the extracted DNA solution were tested. After passing the test, the sample was stored in the refrigerator at −20°C. (2) Selection method of SNP loci. Two tagSNPs of rs13306146 and rs521674 were selected in this study. Briefly, the two SNPs are located in the 5′-promoter and 3′-untranslated regions. The tagSNPs were selected by Haploview (version 4.2) using pairwise linkage disequilibrium with default settings (the Hardy–Weinberg *P*-value cutoff was 0.05, and an *r*^2^ value > 0.8). For the selected SNPs, primers were designed with the help of the PyroMark Assay Design 2.0 (Qiagen, Hilden, Germany) and amplified by PCR (Eppendorf, Hamburg, Germany), the selected loci being classified by pyrosequencing (PyroMark Q24, Qiagen, Germany) using PyroMark PCR Kit (Qiagen, Germany); the reaction reagents include DNA polymerase, ATP sulfurytase, luciferase, and apyrase.

### Dual Luciferin Reporter Gene Assays

The dual luciferase reporter gene containing 100 bp before and after the rs13306146 site was constructed. The *α_2A_AR* rs13306146 G allelic reporter constructs were prepared by site-directed mutagenesis technique. HeLa cells were cultured in 24-well plates, and reference allele (A) or alternative allele (G) α_2A_R plasmids were separately transfected or co-transfected with miR-646 mimics or negative control (NC) mimics using Lipofectamine 2000. Forty-eight hours after transfection, firefly, and Renilla luciferase activities were measured using the Dual-Luciferase^®^ Reporter Assay System (Promega) (Linnstaedt et al., 2017; Huang et al., 2020).

### QRT-PCR of mRNA

Total RNA was extracted using TRIzol, with cDNA synthesized with PrimeScript^TM^ RT reagent Kit (TaKaRa, Bio. Inc., Shiga, Japan). Gene expression was assessed by qRT-PCR using TB Green^®^ Fast qPCR Mix (TaKaRa, Bio Inc.) assay kits. The real-time PCR was performed using the Roche LightCycler 480 PCR System.

### Western Blot Analysis

Total proteins of cells were collected in RIPA lysis and separated by sodium dodecyl sulfate-polyacrylamide gel electrophoresis (SDS-PAGE) and then transferred onto PVDF membrane (Millipore, Bedford, MA, United States). This was then incubated in a blocking solution (5% nonfat milk) and probed with the primary antibody at 4°C overnight. The rabbit polyclonal anti-α_2a_ adrenergic receptor antibody used in the study was purchased from Cell Signaling Technology, and beta-actin (Sigma, St. Louis, MO, United States) was used as a loading control.

### Statistics and Analysis

All statistical analyses were performed using the SPSS 18.0. Continuous variables are presented as mean ± standard deviation. Qualitative variables are described by frequencies and percentages. The comparison between groups was conducted by *t*-test, ANOVA, or chi-squared test (selected according to the data type). The Hardy–Weinberg balance test was performed for all SNP loci, and the chi-square test was used to analyze the association between PDS and non-PDS with SNP loci. The Hardy–Weinberg equilibrium was evaluated by the chi-square test. Binary logistics regression was used to analyze the association between the various factors and PDS. *p* < 0.05 was considered significant.

## Results

### Depression Incidence After Cesarean Section Compared to General Maternal Clinical Data

This study included a total of 568 parturient cases. The total number of participants classed with PDS following the cesarean section was 103, giving a PDS incidence of 18.13%. The clinical characteristics of PDS, vs. non-PDS, following the cesarean section are summarized in Table 1. Comparative differences in PDS, vs. non-PDS, are evident in regard to stress during pregnancy, the relationship between mother-in-law and daughter-in-law, spousal relationship, domestic violence, antenatal emotions, antenatal self-harm ideation, and stressful life events (*p* < 0.05). Elevated pressure during pregnancy, poor relationship between mother-in-law and daughter-in-law, domestic violence, antenatal depressive symptoms, self-harm ideation, and stressful life events were significantly increased in women with PDS. No significant differences between the two groups were found for other clinical characteristics.

**TABLE 1:** Clinical characteristics of all participants.

Variables		PDS	Non-PDS	*p*	OR (95% CI)
Age	≥35 Years	19 (20.4%)	74 (79.6%)	0.530	1.20 (0.68–2.08)
	<35 Years	84 (17.7%)	391 (82.3%)		
Primipara	No	66 (19.6%)	269 (80.4%)	0.252	1.30 (0.83–2.01)
	Yes	37 (15.9%)	196 (84.1%)		
Conception method	Natural	93 (17.7%)	432 (82.3%)	0.365	0.71 (0.34–1.49)
	Artificial	10 (23.3%)	33 (76.7%)		
Full-term pregnancy	Yes	94 (18.0%)	427 (82.0%)	0.850	0.93 (0.44–1.99)
	No	9 (19.1%)	38 (80.9%)		
Single birth	No	7 (15.2%)	39 (84.8%)	0.592	0.80 (0.35–1.84)
	Yes	96 (18.4%)	426 (81.6%)		
Stress during pregnancy	Severe	19 (29.2%)	46 (70.8%)	5.3 × 10^–5^	
	Moderate	56 (24.6%)	184 (76.4%)		
	Mild	28 (10.6%)	235 (89.4%)		
Spousal relationship	Good	84 (16.7%)	418 (83.3%)	0.046	
	Moderate	17 (27.9%)	44 (72.1%)		
	Poor	2 (40.0%)	3 (60.0%)		
In-law relationship	Good	68 (15.3%)	377 (84.7%)	0.004	
	Moderate	33 (28.4%)	83 (71.6%)		
	Poor	2 (28.6%)	5 (71.4%)		
Domestic violence	No	96 (17.3%)	458 (82.7%)	0.007^&^	
	Cold violence	7 (53.8%)	6 (46.2%)		
	Physical violence	0 (0.0%)	1 (100%)		
Education	High school degree or below	40 (20.9%)	151 (79.1%)	0.099	
	Bachelor degree	60 (18.0%)	274 (82.0%)		
	Master degree or above	3 (7.0%)	40 (93.0%)		
Economic status (RMB/month)	>10,000	33 (16.3%)	169 (83.7%)	0.393	
	2,500–10,000	69 (19.5%)	284 (80.5%)		
	<2,500	1 (7.7%)	12 (92.3%)		
Antenatal depressive symptoms	Yes	27 (49.1%)	28 (50.9%)	3.6 × 10^–10^	5.54 (3.10–9.92)
	No	76 (14.8%)	437 (85.2%)		
Antenatal self-harm ideation	Yes	4 (80.0%)	1 (20.0%)	3.0 × 10^–4^	18.74 (2.07–169.54)
	No	99 (17.6%)	464 (82.4%)		
Stressful life events	Yes	5 (55.6%)	4 (44.4%)	0.003	5.88 (1.55–22.29)
	No	98 (17.5%)	461 (82.5%)		

*^&^Calculated with Monte Carlo.*

### Association Analysis Between Depression Incidence and α_2A_AR Polymorphism

The two SNPs of *α_2A_AR* (rs13306146 and rs521674) were detected by the gene-sequencing technology. The genotype frequencies of the SNPs are listed in Table 2. Distributions of these α_2_AR gene SNPs conform to the Hardy–Weinberg equilibrium (*p* > 0.05). As shown in Table 2, PDS incidence in each genotype of rs13306146 at the SNP of the *α_2A_AR* was different (*p* < 0.05). Further analysis showed that the wild-type homozygous AA of rs13306146 in *α_2A_AR* had an increased incidence of depression, vs. the heterozygous AG and mutant homozygous GG (*p* < 0.05).

**TABLE 2:** Correlation between *α_2A_AR* gene polymorphisms and postpartum depressive symptoms (PDS).

					Models					
Gene	SNPs	Genotype (DD/Dd/dd)	PDS	Non-PDS	Additive		Dominant		Recessive	
					OR (95% CI)	*p*	OR (95% CI)	*p*	OR (95% CI)	*p*
*α_2A_AR*	rs13306146	AA	57 (55.3%)	185 (39.8%)	Reference	0.013*	0.533 (0.347–0.820)	0.004*	0.579 (0.268–1.253)	0.161
		AG	38 (36.9%)	221 (47.5%)	0.558 (0.354–0.879)	0.011*				
		GG	8 (7.8%)	59 (12.7%)	0.440 (0.199–0.975)	0.039*				
	rs521674	TT	42 (40.8%)	213 (45.8%)	Reference	0.649	1.228 (0.796–1.893)	0.353	1.089 (0.543–2.183)	0.810
		TA	50 (48.5%)	206 (44.3%)	1.231 (0.783–1.936)	0.368				
		AA	11 (10.7%)	46 (9.9%)	1.213 (0.581–2.533)	0.607				

*p < 0.05. Additive: DD vs. Dd vs. dd; Dominant: DD vs. Dd + dd; Recessive: DD + Dd vs. dd.

### Logistic Regression Analysis of Risk Factors Related to Postpartum Depression

Factors whose frequency is no less than 5% of the sample size with differences between PDS and non-PDS groups were considered as potential confounding variables in the regression analysis. Finally, stress during pregnancy and antenatal depressive symptoms were included in logistic regression analysis, with expansion coefficient (VIF) both of 1.048, which showed no collinear relationship between them.

The results of the binary logistic analysis using forward (LR) are presented in Table 3, showing that the final factors to enter the model were stress during pregnancy [*p* = 0.002, OR (95% CI) = 1.67 (1.21–2.32)], antenatal depressive symptoms [*p* = 1 ×10^–6^, OR (95% CI) = 4.59 (2.49–8.45)], and α_2A_AR rs13306146 polymorphism [*p* = 0.006, OR (95% CI) = 0.61 (0.42–0.87)]. After false discovery rate (FDR) correction, we still observed significance for rs13306146 polymorphism (*P*_*FDR*_ = 0.012). The results of binary logistic regression showed that prenatal depression, high stress during pregnancy, and pregnant women with *α_2A_AR* rs13306146 AA were all important risk factors for PDS after cesarean section.

**TABLE 3:** The association of rs13306146 and PDS with logistic analysis.

Variable	VIF^#^	B	S.E.	Wals	*p* value	OR (95% CI)
Stress during pregnancy	1.048	0.514	0.167	9.525	0.002*	1.672 (1.206–2.318)
Antepartum depression	1.048	1.524	0.312	23.924	1 × 10^–6^*	4.590 (2.492–8.454)
rs13306146	1.000	–0.503	0.182	7.587	0.006*	0.605 (0.423–0.865)

*p < 0.05. ^#^The expansion coefficient of variance inflation factor (VIF) with collinearity diagnostics.

### The rs13306146 A > G Polymorphism Influences α_2A_AR Expression Levels

**FIGURE 1:**
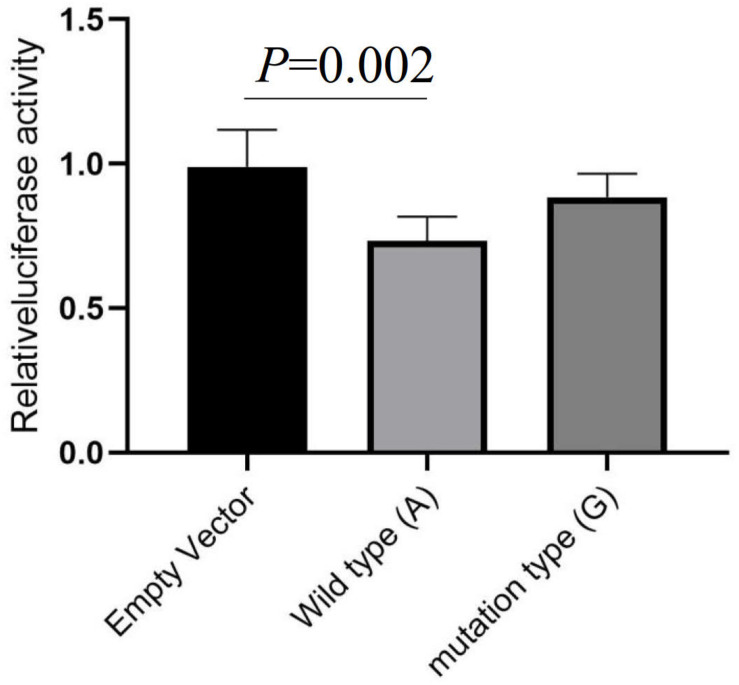
Luciferase reporter assay evaluates the effect of rs13306146 on *α_2__*A*_*AR transcriptional activity. The A allele of rs13306146 decreases *α_2__*A*_*AR transcription activity in HeLa cell. Data were presented as mean ± SD with the replication of *n* = 3.

The Encode database predicated that rs13306146 has candidate *cis*-regulatory elements (ccREs), which may be involved in regulating *α_2A_AR* gene expression levels. In this research, the transcriptional activity was studied by the method of dual luciferin reporter gene, with the recombinant plasmid containing the rs13306146 A or G allele. We showed that compared with the empty vector, the transcriptional function of the *α_2A_AR* rs13306146 reference allele (A) was significantly decreased, while the fluorescence activity of the G allele carrier is similar to that of the empty carrier (Figure 1). The results suggest that the individual carrying the *α_2A_AR* A allele had low transcriptional activity of the *α_2A_AR* gene.

**FIGURE 2:**
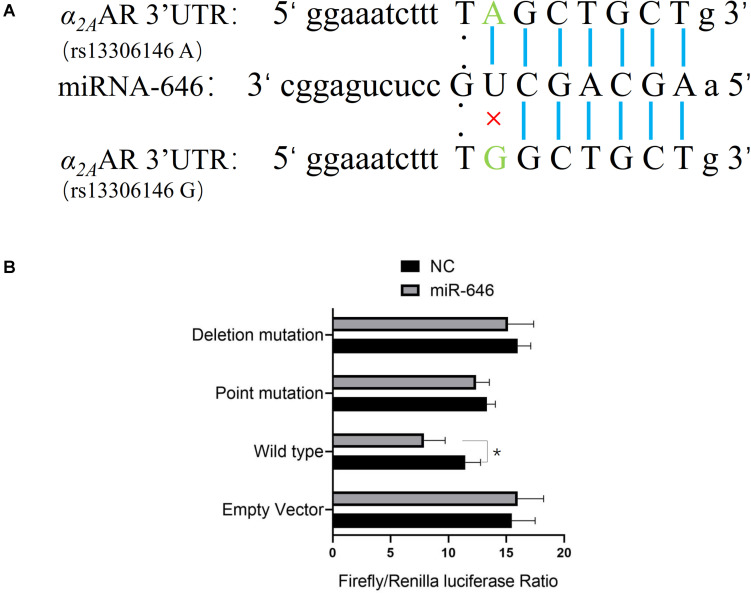
miR-646 directly targets the *α_2__*A*_*AR rs13306146 A allele. **(A)** Bioinformatics analysis of potential miR-646 binding to *α_2__*A*_*AR rs13306146 polymorphisms. **(B)** Luciferase assay. HeLa cells were co-transfected with miRNA mimics or pMIR-Report (vector) of reference allele (A) and alternative allele (G) of *α_2__*A*_*AR 3′UTR. Firefly luciferase signals were normalized with Renilla luciferase signals. The Renilla luciferase activity of each sample was normalized to the firefly luciferase value and plotted as relative luciferase activity. Data were presented as mean ± SD with the replication of *n* = 3. *p < 0.05.

**FIGURE 3:**
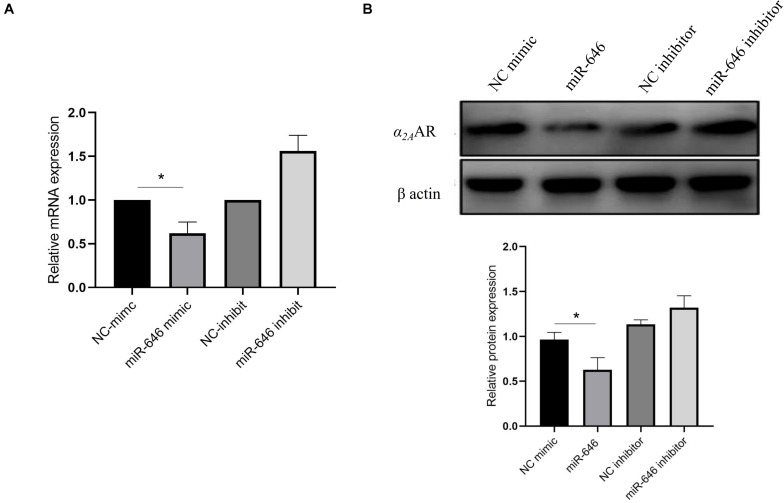
miR-646 inhibited α2AAR expression. MiR-646 significantly decreases α2AAR mRNA **(A)** and protein **(B)** expression in HeLa cell. The values (mean ± SD from three independent experiments) are relative to NC which was set as 1. *p < 0.05.

Further analysis of the rs13306146 site showed the SNP to be located in the *α_2A_AR* 3′UTR region, a region that is most likely to be regulated by microRNAs (miR). Through the online database miRNASNP2^1^, we predicted that the rs13306146 polymorphism of *α_2A_AR* 3′UTR may change the binding ability of miR-646, with the rs13306146 G allele leading to a loss of miR-646 regulation of the *α_2A_AR* gene (Figure 2A). Following a double fluorescein reporter gene experiment, results indicate that miR-646 could significantly downregulate the fluorescence activity of *α_2A_AR* rs13306146 A > G reference allele (A) reporter gene vector, but had no significant effect on the fluorescence activity of the alternative allele (G) vector (Figure 2B). It is proposed that miRNA-646 can bind to the 3′UTR region of the *α_2A_AR* gene to inhibit the transcription of the *α_2A_AR* gene. As such, the *α_2A_AR* rs13306146 polymorphism can significantly affect the mRNA expression of *α_2A_AR* gene by miR-646 (Figure 3A). Western blot-derived data also show miRNA-646 to downregulate *α_2A_AR* expression levels (Figure 3B).

## Discussion

Postpartum depressive symptoms is a serious disorder, with implications for the mother, neonate, and wider family. This study investigated PDS risk factors and the relationship between PDS and NE receptor SNPs. The results showed that (1) *α_2A_AR* rs13316046 polymorphism was associated with PDS. (2) PDS risk in women with the *α_2A_AR* rs13316046AA genotype was significantly higher vs. women with AG and GG genotypes. (3) The effect of *α_2A_AR* rs13316046 A > G polymorphism on PDS may be due to the change in the binding ability of miR-646 at *α_2A_AR* 3′UTR with A > G, thereby affecting α_2A_AR transcription levels. (4) Increased stress during pregnancy, poor relationship between mother-in-law and daughter-in-law, spousal relationship, domestic violence, antenatal depression, self-harm ideation, and stressful life events were all associated with increased PDS incidence.

Norepinephrine neurons are a component of the diffuse modulatory systems in the central nervous system (Castelino and Ball, 2005). Although the distribution of central NE neurons is scattered across different brain regions, the highest concentration of NE neurons is in the locus coeruleus, although there is also evidence of NE nuclei in the hippocampus, frontal cortex, lateral reticular nucleus, and other brain regions (Borodovitsyna et al., 2017). Being associated with arousal, including the sympathetic nervous system, NE dysfunction has long been associated with regulation by stress, and thereby with depression (Montoya et al., 2016). *α_2A_AR* is an important receptor of central noradrenergic nervous system self-regulation. The results of the current study demonstrate a role for *α_2A_AR* polymorphism in PDS, with PDS incidence in women carrying the *α_2A_AR* rs13306146 AA genotype being significantly higher than in those carrying the *α_2A_AR* rs13306146 AG or GG genotype. These data suggested that NE and α_2A_AR genetic variations were associated with PDS.

To explore the effect of the *α_2A_AR* rs13306146 A > G polymorphism on *α_2A_AR*, its expression level was studied by the dual luciferin reporter gene method. The results showed that the transcriptional function of the *α_2A_AR* rs13306146 reference allele (A) was lower than that of the alternative allele (G), and the α_2A_AR expression level was correspondingly decreased. This suggested that the effect of *α_2A_AR* rs13306146 A > G polymorphism may reduce α_2A_AR expression. It is worth noting that α_2A_AR not only has self-regulatory effects on central NE but also has varying degrees of regulatory effects on the serotonergic and glutamatergic neuronal systems, also playing a major role in the CNS response to external stress (Drago et al., 2011). Stress is the main environmental risk factor in depression pathogenesis (Hammen, 2018), which was supported in the current study indicating that stress during pregnancy is an important PDS risk factor. Considerable evidence shows that the locus coeruleus α_2A_AR can inhibit stress-induced depression-like behavior (Wang B. et al., 2017), while α_2A_AR knockout mice show depression-like behavior that does not respond to the NE reuptake inhibitor, imipramine (Schramm et al., 2001). Animal experiments show stress to increase central NE system activation, including downregulating brain α_2A_AR levels following acute stress, while upregulating α_2A_AR levels in the hypothalamus, cortex, hippocampus, and other brain regions following chronic stress (Flügge et al., 1997).

As α_2A_AR is important to the presynaptic negative feedback control of NE release, the results of the current study show that the effect of *α_2A_AR* polymorphism on transcriptional function and PDS incidence is of some importance. We propose that the PDS susceptibility associated with the *α_2A_AR* rs13306146 AA genotype arises from transcriptional *α_2A_AR* inhibition, with consequences for central NE regulation of the locus coeruleus, hypothalamus, cortex, hippocampus, amygdala, and other brain regions, including their involvement in autonomic nervous system function under stress. This genotype will increase neuronal responses to excitatory/stressful stimuli, thereby enhancing depression susceptibility. Such proposed mechanisms are supported by the significant reduction in PDS incidence following dexmedetomidine administration to stimulate α_2_ adrenergic receptors, as shown in our previous work (Yu et al., 2019). We further explored the mechanism of the effect of *α_2A_AR* rs13306146 A > G gene polymorphism on α_2A_AR gene transcription. Using bioinformatics analysis, we found that there were transcriptional regulatory binding elements in this site, with A vs. G, the allele of rs13306146 dramatically decreasing α_2A_AR transcription activity. Further, results show that miR-646 can bind to the 3′UTR region of the α_2A_AR gene to inhibit α_2A_AR gene transcription. Being a class of endogenous noncoding small RNAs containing approximately 20–24 nucleotides, miRNAs post-transcriptionally regulate gene expression, with individual miRNAs able to regulate up to 100 genes. Given such impacts on patterned gene expression, miRNAs are involved in a variety of biological processes, including early development, cell proliferation, differentiation, and apoptosis (Bhaskaran and Mohan, 2014; Lu and Rothenberg, 2018), with miR-146a and miR-212 previously shown to be altered in the monocytes of PDS women (Weigelt et al., 2013). However, the current study is the first to investigate miR-646 in PDS. As with most miRNAs, almost all previous studies have been carried out on tumor cells (Pan et al., 2016; Niu et al., 2018). Investigation on the role of miR-646 in non-neoplastic CNS cells will be important to determine the coordinated changes that may be occurring with changes in α_2A_AR regulation. The results of the current study strongly support a role for genetic and stress-dependent epigenetic interactions in PDS pathogenesis. To date, many studies have shown an association between stress during pregnancy stress and PDS (Bernazzani et al., 1997; Da Costa et al., 2000; Rogathi et al., 2017). The current study also shows roles for genetic factors, domestic violence, prenatal stress, and prenatal emotion in PDS pathophysiology, which is consistent with our previous research in this area. Although non-interventional genetic factors are relevant, many PDS risk factors can be more readily targeted for prevention and treatment. Psychological and emotional states that contribute to stress-driven pathophysiological changes in PDS are important to monitor and treat prenatally as well as postnatally.

The study has a number of limitations. First, we only focused on women having a cesarean delivery. Future work will determine the generalizability of these results in women having a vaginal delivery. Second, it is also of note that PDS onset may be the result of a combination of genetic susceptibility and environmental factors, which could only be analyzed in studies with larger samples that allow for stratified analysis.

Overall, this study showed a number of factors to be associated with PDS following a cesarean. As well as high stress during pregnancy, severe domestic violence, and prenatal depression, women with the rs13306146 AA genotype of the 3′UTR region of the *α_2A_AR* gene have an increased PDS risk, which is proposed to be mediated by alterations in the binding ability of miR-646, thereby acting to differentially regulate α_2A_AR gene transcription. This is likely to have a number of consequences, including changes in sympathetic nervous system activity, as well as interacting with the array of stressors contributing to PDS risk. Consequently, the study provides insight into the pathophysiology of PDS, also indicating pathways for novel treatment interventions. It is worth noting that we found a significant association of α_2A_AR rs13306146 polymorphism and PDS risk. Genotypes of α_2A_AR may be novel and useful biomarkers for PDS.

## Data Availability Statement

The original contributions presented in the study are included in the article/supplementary material, further inquiries can be directed to the corresponding author.

## Ethics Statement

The studies involving human participants were reviewed and approved by the Ethics Committee of the Third Xiangya Hospital of Central South University. The patients/participants provided their written informed consent to participate in this study.

## Author Contributions

KD and SW wrote the manuscript. KD, CF, and SW conceived and designed the experiments. SQY, STY, JX, HC, GL, LZ, and MP performed the experiments. KD, CF, ZL, and SW analyzed the results. All authors contributed to the intellectual content and commented on the manuscript.

## Conflict of Interest

The authors declare that the research was conducted in the absence of any commercial or financial relationships that could be construed as a potential conflict of interest.

## Footnotes

1 https://compbio.uthsc.edu/miRSNP/miRSNP_detail_all.php

## Abbreviations

PDS: postpartum depressive symptoms
EPDS: Edinburgh Postnatal Depression Scale
NE: norepinephrine
AR: α -adrenoceptors
HMPG: 4-hydroxy-3-metoxyphenyl-glycol
LPS: lipopolysaccharide
SNP: single nucleotide polymorphisms
MAO: metabolic enzymes monoamine oxidase
COMT: catechol-*O*-methyltransferase
ASA: American Society of Anesthesiologists
EDTA: ethylenediamine tetraacetic acid
ECG: electrocardiography
SDS-PAGE: sodium dodecyl sulfate-polyacrylamide gel electrophoresis
ccREs: *cis*-regulatory elements.
